# Strategies for Implementing a One Welfare Framework into Emergency Management

**DOI:** 10.3390/ani11113141

**Published:** 2021-11-03

**Authors:** Hayley Squance, Carol MacDonald, Carol Stewart, Raj Prasanna, David M Johnston

**Affiliations:** 1 Joint Centre for Disaster Research, Massey University, Wellington 6021, New Zealand; c.stewart1@massey.ac.nz (C.S.); r.prasanna@massey.ac.nz (R.P.); d.m.johnston@massey.ac.nz (D.M.J.); 2 Independent Researcher, Masterton 5810, New Zealand; carol.macdonald59@gmail.com; 3 College of Health, Massey University, Wellington 6021, New Zealand

**Keywords:** One Welfare, animals in disasters, implementation, emergency management, animal welfare

## Abstract

**Simple Summary:**

During emergencies, people’s decision-making and actions are strongly influenced by their relationship with their animals. In emergency management, a holistic approach is needed which recognises the important interrelationships between animal welfare, human well-being, and the physical and social environment. It is also vital to break down barriers of collaboration between individuals, organisations, and the community. One Welfare, a concept with human–animal-environment interdependencies at its core, provides a framework to achieve this. Successful implementation of a transformative change will require positive strategies to deal with challenges and to ensure that animals are truly integrated into emergency management, not just included as an aside.

**Abstract:**

Responding to emergencies requires many different individuals and organisations to work well together under extraordinary circumstances. Unfortunately, the management of animal welfare in emergencies remains largely disconnected from emergency management overall. This is due predominately to professional silos and a failure to understand the importance of human–animal-environment (h-a-e) interdependencies. One Welfare (OW) is a concept with these interrelationships at its core. This paper argues that by adopting an OW framework it will be possible to achieve a transdisciplinary approach to emergency management in which all stakeholders acknowledge the importance of the h-a-e interdependencies and work to implement a framework to support this. Acknowledging that such a transformational change will not be easy, this paper proposes several strategies to overcome the challenges and optimise the outcomes for animal welfare emergency management (AWEM). These include legislation and policy changes including h-a-e interface interactions as business as usual, improving knowledge through interprofessional education and training, incorporating One Welfare champions, and recognising the role of animals as vital conduits into communities.

## 1. Introduction

In many countries animals are increasingly included in emergency management legislation and policy with specific organisations delegated responsibility for animal welfare in emergencies, including the development of animal inclusive emergency management plans and is termed AWEM. [1,2,3,4,5,6]. Despite this progress, issues persist, such as, animal welfare response being disconnected from the official overall emergency response [7]. Inadequate AWEM responses often result in increased risk behaviours of animal owners [7]. This can then result in failure to meet the intended outcomes of protecting public safety and wellbeing, animal welfare, food security, and biosecurity [1,4,7,8,9]. Improving AWEM requires a shift from an inclusive approach to one in which animals are fully integrated into emergency management [7]. This demands a transdisciplinary approach to emergency management in which all stakeholders acknowledge the importance of the h-a-e interdependencies and implement a framework to support this [10]. Terms such as multidisciplinary, interdisciplinary, and transdisciplinary are frequently used interchangeably to discuss efforts that involve several disciplines. A multidisciplinary approach draws on the knowledge of different disciplines, but disciplines that work independently considering an issue, and their perspectives typically remain unchanged. With an interdisciplinary approach, knowledge is shared between disciplines but work and perspectives continue to be largely rooted in independent disciplines. A transdisciplinary approach involves diverse stakeholders providing complementary perspectives and contributing unique expertise to search for ‘whole of problem’ solutions that ‘transcend’ their own discipline [11,12].

OW is a concept that describes the interrelationships between animal welfare, human wellbeing, and the physical and social environment with the aim of creating a platform to enhance the understanding of, and response to, the complexities of the h-a-e interrelationships [13,14]. OW acknowledges that h-a-e interrelationships transcend the expertise and boundaries of any one organisation and seeks to transition from traditional management by individual sectors towards an interdisciplinary approach [15,16,17]. It is a way of breaking down barriers between agencies, individuals, sectors, and the community [18]. 

OW evolved from the One Health (OH) concept of structured collaboration and coordination between multiple health science professions to attain optimal human, animal, and environment health systems [19,20,21,22,23]. OH has been cited as an efficient and sustainable governance approach to address complex health issues [16]. It is argued that although OH has sparked an evidence-based body that goes beyond individual disciplines, the strong health focus lacks a vision of a set of social, cultural, economic, and environmental outcomes whose interdependence is similarly acknowledged, thus creating a significant gap in welfare-focused approaches [14,24]. To address this gap, OW was developed as a broader concept to embrace an interdisciplinary approach [21,25,26]. OW is better suited to the emergency management context as it is not solely focused on health and, by encompassing social, economic, environmental, and cultural interdependencies, is more holistic.

While little is known about how the new OW concept might be applied in practice, the experience from OH implementation provides valuable insights to inform the implementation of an OW framework in emergency management. Although positive steps towards achieving an integrated OH approach have occurred over several decades, there continue to be challenges in its implementation [16,19,24,27,28]. These include lack of awareness and understanding of OH across the sectors required to implement the approach, insufficient “whole-of-government” policy prioritisation and funding to support OH, lack of integrated education and training programmes, reliance on the leadership of a few individuals and institutions, and the translation of global OH to local-level efforts and outcomes [16,18,19,24,27]. Dos S. Ribeiro et al. explain: “the challenges in stakeholder collaboration relate to the fact that a multidisciplinary team of scientists [and practitioners] working together but within their own silo is not enough for the knowledge co-creation proposed in OH innovations” [24] (p. 3).

OW provides a framework to address emergency management silos that negatively impact on AWEM, but challenges, similar to those faced with OH, are likely. Strategies will be needed to address these, and to provide practical examples and evidence of the value of OW. While OW has been mentioned in AWEM literature, this has largely been limited to comments about the need to include animals by considering them alongside people [8,14,29,30]. There has been no detailed consideration of the potential for OW to promote interdisciplinary collaboration through a transdisciplinary approach. This paper addresses that gap by arguing for the full integration of animals in emergency management and discusses how that could be implemented.

**Figure 1:**
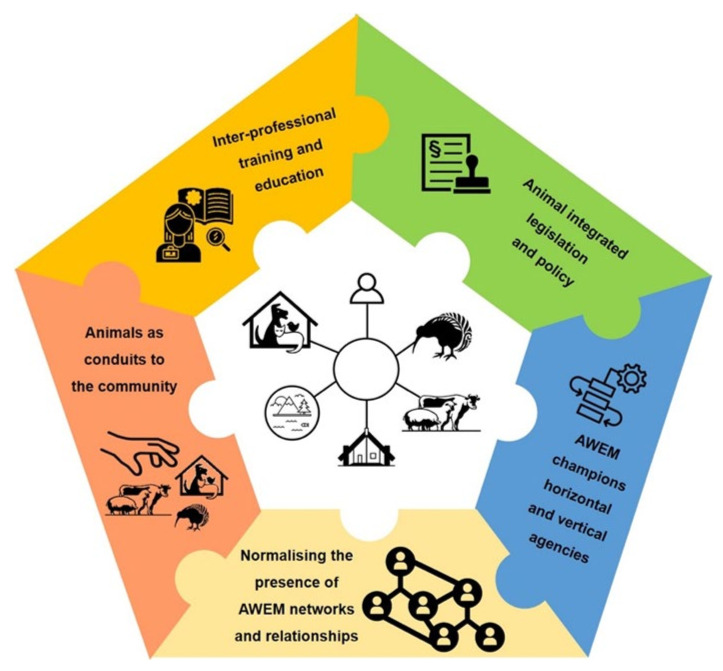
Implementation strategies for OW in Emergency Management through an animal lens.

Additionally, this paper synthesises and extends a suite of conceptual frameworks, scholarly work around implementation, research findings, and practice-based knowledge into an integrative framework (Figure 1). Figure 1 illustrates several core strategies aligned with the complex interdependencies between humans, all animals (companion, production, and wildlife), and the social (the social environment is depicted as a marae, the social and spiritual hub of a Māori community, a place where people gather for meetings, celebrations, funerals, and other important events) and physical environments. The strategies that have been tested and refined within the New Zealand emergency management system include legislation and policy changes including: h-a-e interface interactions as business as usual, improving knowledge through interprofessional education and training, incorporating OW champions, and recognising the role of animals as vital conduits into communities.

## 2. Legislation and Policy: From Animal-Inclusive to Integrated

In New Zealand and elsewhere, the failure of emergency management legislation and policy to reflect the h-a-e interdependency has artificially compartmentalised human, social, and animal welfare and created barriers to collaborative practice [7,13,31,32]. However, given that community wellbeing and safety is at the heart of emergency management, it is vital to break down the sectoral partitioning that exists between emergency management agencies, emergency service organisations, AWEM support agencies, and other sectors of activity.

Animal welfare legislation, separate from emergency management and emergency service legislation, has limited application when it comes to collective planning for and management of animal welfare in disaster situations [33]. As a result, fragmented policies and plans set different goals and standards for various agencies, sometimes in direct conflict. 

For example, law enforcement agencies have the power to temporarily close a road by placing cordons, if they believe that there is a danger to the public. However, cordons may be counterproductive if animal owners engage in risky behaviours, such as breaking cordons to gain access to their animals [4]. Separating owners from their animals also means that they are unable to fulfil their duty of care (providing food, water, and shelter) as required under animal welfare legislation [8,33]. Without consultation, the wider consequences of measures, such as the placement of cordons, are not generally factored into the decision-making.

If emergency management legislation and policy is to be fit-for-purpose, it must have multilateral coherence with other relevant legislation, be consistent across Acts and policy, and reflect h-a-e interdependencies to enhance social protection [18,34,35]. Generally, however, legislation and policy are not agile enough to reflect the complex needs of communities with changes being made in a reactive manner, rather than proactively, which only serves to increase sector fragmentation [27,34,35,36].

Legislation and policy must respond to evolving societal needs and foster a holistic approach to emergency management that acknowledges h-a-e interdependency. This can only be achieved when animals are not simply ‘included’ in emergency management legislation and policy, but are fully integrated and done so in a manner that reflects a “whole-of-person” and “whole-of-society” approach [7,10,12,22,27,34,37,38]. For example, the insistent call for animal welfare plans to be in place as a mechanism to protect animals in emergencies is problematic. Plans are only of value if they are co-created with stakeholders who experience the h-a-e interface, build capacity and capability, and integrate h-a-e across all pillars of emergency response and recovery. For example, an animal plan that includes animal evacuation considerations will be constrained and ineffective if the overall evacuation plan does not directly reference animals or is not aligned with the animal plan. By focusing solely on animals, silos are created and the intended outcome of protecting public safety, animal welfare, the economy, and biodiversity is not achieved. 

Legislation and policy must reflect a transdisciplinary view of emergency management in which h-a-e interdependencies are an integral component. This is a critical step in addressing interagency fragmentation, competition, and breaking down barriers created by the bureaucratic division of responsibility, which is counter-productive to the desired goals of emergency management.

Flexible, innovative practices implemented during the Eastern Bay of Plenty flood [7] and the 2019 Pigeon Valley wildfire responses [39] in New Zealand illustrate the positive potential of an OW approach to emergency management. During these emergencies, an animal welfare coordinator was included in the emergency operations centre (EOC) and incident management team (IMT), which assisted with better integration of the animal welfare response with an overall better response, facilitated access to valuable information and intelligence, offered opportunities to advocate for the inclusion of animals in decision-making, and articulated the consequences of decision-making on animal welfare and animal owner behaviour. An animal welfare coordinator also became part of the cordon management team, which created opportunities for amalgamated animal response teams to be granted emergency access to cordoned areas to assess animals and address any immediate needs. The inclusion of multiple and more diverse perspectives within pivotal decision-making teams, such as the EOC, was a departure from previous practice and illustrates how the OW approach can serve as a catalyst to transdisciplinary consequence management [40]. In this way, response and recovery environments provide a unique opportunity to test, refine, and develop ideas and provide practice-led evidence in support of legislative and policy changes.

## 3. Normalising the Presence of h-a-e Interface Networks and Relationships

Emergencies are complex and fluid. They require a diversity of people, disciplines, and organisations to share expertise, perspectives, and resources, and to collaborate and form a common goal of addressing acute challenges facing communities [41]. Strong collaborative networks based on existing, trusted relationships support better decision-making processes and actions during an emergency [42]. Yet, many of those encountering the human–animal interface during an emergency meet for the first time in a highly stressful environment which is not conducive to developing the interprofessional trust and understanding necessary for positive transdisciplinary relationships [2,7,43,44].

In the absence of pre-existing relationships, emergency management organisations are frequently required to act as brokers between agencies during a response [45]. However, networks created under such conditions are rarely long-lasting, resilient, or cost-effective with previous siloed ways of working coming to the fore [46]. Ideally, organisations need to forge direct links with one another so that their interactions are normalised into business-as-usual activities, rather than relying on emergency management organisations to broker collaborative networks during emergencies. In this way, business-as-usual interactions can lead to more positive and trusting relationships and networks that underpin meaningful collaboration during times of crisis.

Disasters, while affecting whole communities, share some impact characteristics with other crises and extreme stressors experienced at the h-a-e interface, such as domestic violence, animal hoarding, structural fires, and animals requiring technical rescue [15,26,47,48,49]. These incidences often transcend the expertise and/or jurisdiction of any one organisation and should involve agencies such as emergency services, law enforcement, human services, animal control, environmental health, animal charities, and veterinary professionals [43,49,50]. In practice, however, agencies generally work in silos, and do not deploy simultaneously, and if they do it is usually ad hoc due to informal individual relationships [7,51]. This siloed approach leads to unsafe practices [7,52,53], inadequate resourcing and capability and, in the case of animal hoarding, a high recidivism rate [17,49,54].

For example, while fire services frequently experience h-a-e interactions with the presence of animals in structural fires, motor vehicle accidents, and with entrapped animals requiring rescue, they do not routinely work with animal organisations. Such incidences occur more frequently than floods, fires, and earthquakes, yet the presence of animals creates the same complexities [48,52,55,56].

Safe and successful resolution of emergency situations involving animals requires emergency services personnel to have specialist training, skills, and equipment, and a collaborative multidisciplinary team [4,48,57,58]. However, emergency services did not traditionally have the skills or experience to respond to the presence of animals in such incidences, have lacked relationships with animal organisations, and relied on an element of luck which resulted in a high risk of serious harm to the animal, owner and responders, and significant reputational risk [44,52]. It is equally important that supporting animal agencies have an understanding of, and align with, emergency management systems and practices [44].

Internationally, the approach to entrapped animal rescue has been increasingly transdisciplinary and collaborative with the inclusion of interprofessional education and training, simultaneous deployment of animal expert resources by emergency services, utilisation of complementary skills and resources, and inclusion of the presence of animals as part of the incident risk assessment [48,52]. This has resulted in better response outcomes with a decreased risk to responders and animal owners, reduced mortality of animals, created a better understanding of individual disciplinary skill sets, roles, and responsibilities, and more efficient and effective responses and opportunities for transdisciplinary teams [48,55,59]. 

Regular interactions between fire services and animal organisations over ‘routine’ incidences would cement the relationships needed during an emergency, such as a wildfire, earthquake, or flood event. This would enable them to move from novel relationships being brokered by emergency management organisations to normalising the relationships needed for AWEM. The transition to collaborative transdisciplinary teams requires a novel approach to identifying and aligning the synergies between an organisations’ core business and h-a-e interdependencies. Focusing on the synergies between diverse positions can improve access to high-quality information and incentives within the network, increase confidence, reliability and integrity, and develop a sense of trust and reciprocity between partners [60].

## 4. Interprofessional Education and Training

A key step in moving emergency management systems from fragmentation to a position of strength and unity is to become a collaborative practice-ready emergency management workforce [41,61]. There is clear evidence that interprofessional education enables effective collaborative practice through attitude change, greater understanding of the roles and responsibilities of others, increased awareness of barriers across professions, and increased awareness of the importance of professional collaboration [61,62,63].

Interprofessional education and training is delivered in health and aviation sectors and is recognised as an essential element of successful transdisciplinary collaborative practice, noting that just working with others in scenarios or temporary teams is not enough to build an effective collaborative practice-ready workforce [16,18,19,22,28,61,62,64,65,66].

Despite individuals being expected to possess the skills, knowledge, and attitudes necessary to work together in interprofessional emergency management teams, education and training has largely occurred in professional silos or assumed to be learned during scenarios [67]. Emergency management and emergency service disciplines are taught almost exclusively in isolation from other emergency responders who experience the h-a-e interface. Animals in emergencies are generally not included in emergency management, emergency services, veterinary, animal science, agriculture, or environmental science curricula [68]. The lack of interprofessional education and poor curriculum integration amplifies differences in organisational cultures and reinforces barriers to effective interagency collaboration.

If collaborative practice is to become the norm, change will be needed in attitudes, systems, and operations. By embedding interprofessional education and collaborative practice into legislation, accreditation requirements and/or registration criteria, policy-makers and government leaders can champion change and endorse interprofessional collaboration [61]. Leaders, who choose to contextualise, commit, and champion interprofessional education and collaborative practice, position their emergency management system to strengthen disaster risk governance, a priority of the Sendai Framework for Disaster Risk Reduction 2015–2030 [69]. Endorsed by the United Nations General Assembly in 2015, the framework aims to substantially reduce disaster risk and losses in lives, livelihoods, and health and in the economic, physical, social, cultural, and environmental assets of people, businesses, communities, and countries [69]. It recognizes that the State has the primary role to reduce disaster risk, but that responsibility should be shared with other stakeholders, including local government, the private sector, and other stakeholders [70].

The potential for working relationships in emergency management to be in flux due to the rise and complexity of emergency events, new occupations, professionalisation of emergency management, and challenges to the historically dominant single profession makes the present an ideal time to promote collaborative practice and implement interprofessional education.

Interprofessional education and training should promote complementary approaches to emergency management and include core interprofessional practice competencies across disciplines whilst being sympathetic to the conceptual and practical differences.

## 5. One Welfare Champions

The shift to an OW emergency management framework will require a significant shift with stakeholders expected to operate in a context of collaborative practice with different organisational cultures coming together and working towards a common goal. The success of such a challenge, requiring commitment over a long period of time [24], is critically influenced by the presence of strong and innovative champions of change [24,35,71,72]. Transformation of new cross-sector ideas and concepts without transdisciplinary champions rarely have the impetus needed for successful implementation [73]. 

Successful champions have the courage to break down barriers, take risks, and broker opportunities for collaborative practice whilst creating engagement and trust among all stakeholders [24]. They exhibit influence, ownership, physical presence during interactions, persuasiveness, and a participative leadership style [72,74,75]. Facilitating a process to improve the current state to a desired future level [2,74,75], champions use their mana (in Māori, mana refers to a person’s prestige, authority, control, power, influence, status, spiritual power, and charisma, https://maoridictionary.co.nz/word/3424, accessed on 1 October 2021), knowledge, resources, and influence to help navigate the complex socio-political maze within their organisations. Using language that their discipline understands, they address resistance to new ideas and build organisational coalitions [72,76].

OW champions will need to work within and across organisations experiencing the h-a-e interface before, during, and after emergencies. This includes all levels of government, emergency services (police, fire), emergency responders (emergency management officers, response teams, lifelines, and utilities), defence forces, human service organisations, veterinary professionals, human and animal welfare charities, primary sector, and geological and environmental practitioners.

Champions for OW must understand the challenges their organisations experience due to h-a-e interdependencies, know how to connect between multiple actors, domains and levels, forge change through transdisciplinary collaborative practice, and be able to shift the focus from business-as-usual to innovative practice. Collectively, champions need to be able to identify and utilise strengths, opportunities, and comparative advantages of disparate disciplines and organisations whilst engendering co-creation central to OW initiatives [24,29,72].

If OW champions, representative of all levels within organisations and all key stakeholders, are engaged from the outset through the entire process, initiatives can be planned, designed, and implemented in a collaborative manner across all sectors so that deeper and sustainable change can be achieved for AWEM and emergency management.

## 6. Animals as a Conduit to the Community

During emergencies, human decision-making and actions are strongly influenced by their relationship with their animals [6,55,56,58]. Given that over 60% of urban and 90% of rural households in developed countries own animals [4,5,6], a failure in emergency management to acknowledge this is a failure to consider the whole person, and the measures intended to protect human life and wellbeing may, in fact, be counter-productive [7].

During disasters, animal attachment can pose a risk to human safety but conversely, it can be leveraged through community engagement strategies to increase disaster preparedness [59,77]. Shifting the balance from the negative influence of the human–animal bond to more positive outcomes will require a transdisciplinary approach to develop innovative strategies to engage with and motivate animal owners to better prepare for and respond to disasters. 

Animal support organisations have a unique opportunity to create connections with people due to their mutual connection with animals. This indirect social benefit of animal ownership can bridge the gap between people and facilitate coordinated, cooperative actions for mutual benefit [15,78]. 

In emergencies, people cannot always express their emotional needs, and innovative engagement strategies are needed to ensure equitable access to available resources [55]. Asking about animals can be an ‘icebreaker’ in a social setting and provides a means of building rapport [36] and trust between newly acquainted people [78]. This mutual, relatively ‘safe’ topic of conversation can elicit important information about relationships and family functioning [79] and can be used to help individuals establish social connections. 

This is particularly true for vulnerable or hard-to-reach members of the community, such as the elderly, people affected by a mental illness or drug dependencies, refugees, indigenous people, women, single parents, people with disabilities, and the homeless [55,80]. Though socially isolated and/or vulnerable individuals may possess the skills to function on a day-to-day basis, they may lack the resilience to cope with a crisis situation. Where connectedness to others within a community may be lacking, the support people feel from animals can strengthen the emotional resilience of socially isolated individuals [81]. 

During the recovery phase of disasters when communities can become fragmented and social support networks are lost, there can be a rise in the number of socially isolated and/or vulnerable people. It is during this time that people need appropriate psychosocial assistance, but many may not know where to seek help or believe that they are not entitled to it [82]. As many animal owners are motivated to look after their animals before themselves, and have high trust and pre-existing relationships with animal service organisations, these organisations play a valuable role in the psychosocial recovery of communities, such as the role of navigators. 

A navigator’s role is to link people to other agencies providing support services and to help people navigate their way through the complex support systems that activate in emergencies, such as financial assistance, temporary accommodation, psychosocial services, and insurance support. Animal welfare support organisations may not be aware of support services available or have training in psychological first aid; therefore, navigators require collaborative multiagency, multidisciplinary support, which includes experts in psychosocial recovery. 

Animal ownership offers a unique opportunity for communicating with people and motivating them to engage in resilience building behaviours that promote survival for themselves and their animals, and facilitate recovery from a disaster. Animal service organisations can act as conduits for hard-to-reach community members and have an important role to play as recovery navigators. It is important to note, however, that as front-line workers in emergencies, those providing animal welfare support may experience mental health consequences as a result of exposures to secondary trauma [83,84,85,86] and may require support themselves. 

## 7. Conclusions

Emergency management requires many different individuals and organisations to work together towards common goals under extraordinary circumstances. Unfortunately, professional silos and a lack of understanding of the importance of h-a-e interdependencies means AWEM remains largely disconnected from emergency management overall. 

This paper argues that by adopting an OW framework and creating a sustained practice of engaging and partnering with others across the h-a-e interface, AWEM responses can be greatly improved. However, as seen with OH, there will be significant challenges moving from theory to practical implementation. To overcome the challenges and optimise the outcomes for AWEM, several key strategies are suggested.

Effective interdisciplinary collaboration is vital if OW is to work in practice, yet current legislation and policy incorporate much that is counter to this. Change at this level is vital and must underscore all other change initiatives. Agencies involved with the human–animal interface during an emergency must be supported to develop positive transdisciplinary relationships through business-as-usual interactions, rather than waiting until a crisis, which is not conducive to building trust and understanding. Interprofessional education and training also has a critical part to play in improving interdisciplinary collaboration by increasing knowledge of the multiple roles and responsibilities within AWEM, and by developing skills for functioning as practice-ready interprofessional teams.

The implementation of an OW framework is transformational change. Engaging committed and skilled change champions representative of all stakeholders and levels within organisations will be essential across all phases of implementation. Finally, the human–animal bond should be harnessed as a valuable conduit for communication and engagement with communities and significant stakeholders central to the OW concept.
